# Age is associated with prognosis in serous ovarian carcinoma

**DOI:** 10.1186/s13048-017-0331-6

**Published:** 2017-06-12

**Authors:** Fei Deng, Xia Xu, Mengmeng Lv, Binhui Ren, Yan Wang, Wenwen Guo, Jifeng Feng, Xiaoxiang Chen

**Affiliations:** 1 Department of Gynecologic Oncology, Nanjing Medical University Affiliated Cancer Hospital, Jiangsu Cancer Hospital, Jiangsu Institute of Cancer Research, 42# Baiziting street, Nanjing, Jiangsu 210009 People’s Republic of China; 2 Department of Chemotherapy, Nanjing Medical University Affiliated Cancer Hospital, Jiangsu Cancer Hospital, Jiangsu Institute of Cancer Research, 42# Baiziting street, Nanjing, Jiangsu 210009 People’s Republic of China; 3 Department of Thoracic Oncology, Nanjing Medical University Affiliated Cancer Hospital, Jiangsu Cancer Hospital, Jiangsu Institute of Cancer Research, Nanjing, Jiangsu 210009 People’s Republic of China; 4 Department of Pathology, The Second Affiliated Hospital of Nanjing Medical University, Nanjing, Jiangsu 210009 People’s Republic of China; 5 Department of Pathology, The University of Texas MD Anderson Cancer Center, Houston, TX 77030 USA

## Abstract

**Purpose:**

The survival duration of elderly patients with epithelial ovarian carcinoma is shorter than that of their younger counterparts. This variation in survival duration is likely attributed to differences in the distribution of histological type or grade, International Federation of Gynecology and Obstetrics (FIGO) staging, and undertreatment, but this observation remains controversial. This study aimed to investigate the biological factors other than selection bias associated with the decreased survival of elderly patients with ovarian carcinoma.

**Methods:**

A total of 314 serous ovarian cancer (SOC) patients from Jiangsu Institute of Cancer Research (JICR, PRC) between 2002 and 2012 were retrospectively analyzed, and 774 cases from MD Anderson Cancer Center (MDACC, USA) between 1992 and 2012 were used for validation. The 8-hydroxy-2′-deoxyguanine (8-OHdG) concentration in leukocyte DNA was evaluated by using commercially available enzyme-linked immunosorbent assay kits, and tissue expression was assayed through immunohistochemistry. The associations between survival durations and covariates were assessed by using a Cox proportional hazards model and by conducting a log-rank test.

**Results:**

Advanced age ≥ 65 years was correlated with high histological grade (*p* = 0.02), performance status (*p* = 0.03), primary treatment (*p* = 0.00), and suboptimal surgery outcome (*p* = 0.04) in SOC patients from JICR. Age, FIGO stage, histological grade, and optimal surgery were independently associated with the progression-free survival (PFS; *p* = 0.03, *p* = 0.03, *p* = 0.02, and *p =* 0.04, respectively) and overall survival (OS; *p* = 0.02, *p* = 0.04, *p* = 0.02, and *p* = 0.02, respectively) of the SOC patients from JICR. The 8-OHdG concentration in the leukocyte DNA was higher in the elderly patients than in the younger cases. The high 8-OHdG concentration in the leukocyte DNA indicated poorer median OS (30.0 months, confidence interval [CI]: 23.5–36.5 vs. 42.8 months, [CI] 38.3–47.2) and PFS (14.6 months, [CI] 11.9–17.2 vs. 18.9 months, [CI] 14.4–23.4) than those of their corresponding counterparts in the SOC patients who achieved a clinical complete response from primary treatment.

**Conclusions:**

Compared with younger cases, elderly patients with SOC were commonly characterized by high tumor grade, poor performance status, and undertreatment. High 8-OHdG concentration in leukocyte DNA was associated with advanced age and poor prognosis in SOC patients.

**Electronic supplementary material:**

The online version of this article (doi:10.1186/s13048-017-0331-6) contains supplementary material, which is available to authorized users.

## Background

Epithelial ovarian cancer (EOC) is the leading lethal gynecological malignancy affecting women worldwide [1–3]. EOC is also generally considered an age-related disease. With 65 years as a demarcation criterion, the incidence of this disease in younger patients is 9.34 per 100,000, and this value is lower than that in older patients whose incidence is 52.7 per 100,000 in the USA [4]. In China, approximately 41,000 cases of EOC are diagnosed every year, and roughly 27,650 women died of this disease in 2011 [5]. Approximately 40% of EOC patients are ≥65 years old, but these women account for at least half of all stage III and IV cases and two-thirds of cancer-related deaths [6–9]. With an aging population, the prevalence of ovarian cancer can be expected to increase. The incidence of EOC also increases with age and alters the demographics of this disease. As such, age-oriented biological factors related to the prognosis of EOC should be investigated [10, 11].

The standard care for patients with EOC involves cytoreductive surgery, which is also known as optimal debulking, and six to eight cycles of frontline chemotherapy, including platinum compound with taxane. Although the survival of patients with EOC has generally improved for the past three decades, this progress has yet to provide benefits for elderly patients. Compared with young age, advanced age has been reported as an adverse prognostic factor influencing EOC. However, contradicting results have been obtained, and the mechanisms underlying this observation are poorly defined [12–15].

Studies on elderly patients with EOC have demonstrated that biased results as indicated by the skewed distribution of characteristics may influence clinical outcomes. Several well-known prognostic factors, such as histological type, histological grade, and International Federation of Gynecology and Obstetrics (FIGO) stage, also change with age [16–20]. In addition, frail elderly patients with EOC are less likely to undergo aggressive debulking surgery and standard doublet chemotherapy [14, 21, 22]. Different patterns of care may be affected by age-related morbidities [23], physician-bias-related chemotherapy delay and dose reduction [24, 25], and patient’s living quality preference [26–28]. While on the contrary, it’s reported that elderly patients can tolerate a standard approach instead of conservative therapy with few side effects when adjusted for performance status [29–32]. It has been suggested the inferior survival of elderly patients to that of younger patients is attributed to changes in tumor biological characteristics and inherent resistance to chemotherapy, although this hypothesis remains controversial [33, 34]. Elderly patients with EOC more likely suffer from early recurrence, recalcitrance, and platinum resistance than younger patients do [35].

Distinct recruitment standards described in different studies vary in terms of FIGO stages, pathological types and grades, molecular characteristics, and disease management. The effect of age independent from the influence of these factors on the oncologic survival is also difficult to determine. Serous ovarian carcinoma (SOC) is an archetypal ovarian cancer. Studies have yet to determine whether age affects the prognosis of this disease. Our study was conducted in a SOC subgroup from Jiangsu Institute of Cancer Research (JICR, People’s Republic of China [PRC]) and MD Anderson Cancer Center (MDACC, USA) to examine prognosis-associated factors based on tumor biological characteristics accompanied with age.

## Methods

### Study population

A retrospective chart review was conducted to identify 314 patients diagnosed with SOC treated at JICR from January 1, 2002 to December 31, 2012. In 82 patients alive at the time of analysis, 44 cases were disease free and 36 cases with disease. A total of 774 SOC cases from MDACC were recruited between January 1, 1992 and February 14, 2012 for validation. A total of 110 and 204 elderly (≥ 65 years) patients were recruited from JICR and MDACC populations, respectively. A multidisciplinary team (MDT) consisting of two gynecologic oncologists, two pathologists, one radiologist, and one medical oncologist was set in JICR to guide clinical management. The recruitment criteria of the present study were as follows: patients with ovarian, fallopian tube, or peritoneal serous cancer histologically confirmed by primary surgical procedure or by core biopsy; patients who underwent primary therapy procedure, including cytoreductive surgery and six to eight cycles of frontline doublet chemotherapy. Carboplatinum (area under the curve = 5–6) and paclitaxel (135–175 mg/m^2^) regime administered every 3 weeks. Neoadjuvant chemotherapy (NAC) was recommended for a low likelihood of achieving optimal cytoreduction or a high perioperative risk profile by the MDT. The number of NAC, the opportunity of interval cytoreduction surgery (IDS), and adjuvant chemotherapy cycles were administered on the basis of the decision of the MDT. The follow-up plan included clinical assessment and serum-marker measurements. Disease progression was confirmed by imaging before initiating second-line chemotherapy. The patient follow-up plan after completion of primary treatment included clinical assessment and serum marker measurement, as mentioned previously [16, 18, 19].

### Clinicopathological characteristics

The clinicopathological data of the patients were reviewed, and the following data were collected: age; races; tumor grade; histology; tumor stage; serum tumor markers, including CA-125, CEA during diagnosis, therapy, and follow-up; serum hemoglobin (Hb) content; serum albumin content; performance status; comorbidities; postoperative complications; chemotherapy regime, courses, and clinical or pathological responses; optimality of cytoreductive surgery; and disease status at the last follow-up. Ascites volume was estimated by ultrasound and confirmed by surgical procedure. Regression was defined by an ascites volume < 500 mL. Surgical staging followed the FIGO system. Optimal cytoreduction was defined as the absence of macroscopic disease on the completion of the surgical procedure.

Overall survival (OS) was defined as the length of time from diagnosis to death or to the last follow-up examination of existing patients. Progression-free survival (PFS) was defined as the time interval from primary treatment where in the patient’s condition did not worsen. Disease progression was evaluated by computed tomography (CT)/magnetic resonance imaging (MRI) or positron emission tomography imaging (PET/CT) of the abdomen and pelvis before initiating frontline or second-line chemotherapy. Clinical response was defined in accordance with the standards of the Response Evaluation Criteria in Solid Tumors [36]. The pathology of all patients was initially reviewed by pathologists from JICR (Hou and Xu). A panel of pathologic markers was routinely measured. This study was approved by the ethics committee of the JICR and MDACC. Written informed consent for the publication of this report and any accompanying images was obtained from each patient.

### 8-OHdG level and expression

#### 8-OHdG concentration in leukocyte DNA assay

For measuring the concentration of 8-OHdG, 100 patients were randomly recruited from ovarian specimen bank and investigated using the method reported previously [37–39]. Similar age distribution was observed in the evaluated 8-OHdG concentration in the leukocyte DNA of 100 patients and the whole study population. DNA from leukocyte DNA was salted out within 1 h of collection from fasting venous whole blood (4 mL, with EDTA added to prevent coagulation). The purity of the DNA sample was then routinely measured by OD260 nm/OD280 nm and OD260 nm/OD230 nm using Eppendorf BioPhotometer Plus (Eppendorf, North America). Qualified DNA was stored at *−*80 °C until assay. Nuclease P1 (15 μL, 6 units, Sigma, USA) and sodium acetate (15 μL, 200 mM) were mixed with the dissolved DNA solution. After being incubated at 37 °C for 30 min, the solution was added to Tris–HCl buffer (15 μL, 1 M, pH 7.4) and alkaline phosphatase (7 μL, 2 units, TAKARA, Shiga, Japan) and then incubated at 37 °C for another 30 min. The mixture was filtered through Millipore Microcon columns at 14000 rpm for 10 min, and 50 μL of digested DNA was transferred to one well of an enzyme-linked immunosorbent assay (ELISA) kit (Highly Sensitive 8-OHdG Check, JaICA, Fukuroi, Shizuoka, Japan). Nanogram per milliliter was the unit used for assay, and then the measured value was converted from ng/mL to 8-OHdG/10^6^ dG on the basis of Halliwell.

#### 8-OHdG expression assay in tissues through immunohistochemistry

Seventy-six available sections confirmed as SOC were obtained from 100 patients who were evaluated for 8-OHdG concentration in leukocyte DNA from JICR. After being mounted on frost-free slides, 3–10 μm sections were routinely deparaffinized in xylene and rehydrated through a series of graded alcohols. After being washed with 1× PBS and endogenous peroxidases, the slides were blocked with 1.5% hydrogen peroxide in 1× PBS at 25 °C for 20 min. Next, the slides were washed three times in 1× PBS for 5 min and incubated in blocking solution (1× PBS with 0.1% Triton X-100, 3% bovine serum albumin) with 5% donkey serum at 25 °C for 10 min. Experimental and control (without primary antibody) slides were incubated at 4 °C for 24 h in blocking solution alone or blocking solution with 8-oxoguanine (1:400, ab64548; Abcam, Cambridge MA) antibody, respectively. Biotin-conjugated secondary antibodies (1:200; Jackson ImmunoResearch, West Grove PA) were added, and the slides were incubated at 25 °C for 30 min and then washed with 1× PBS three times. An ABC Peroxidase Staining kit (1:100 dilution of each Reagent A and B in 1× PBS, 32,020; Thermo Scientific, Rockford IL) was applied at 25 °C for 30 min, and the slides were washed with 1× PBS three times. Staining was visualized with peroxidase-sensitive Sigmafast 3,3′-diaminobenzidine tablets (DAB; Sigma, St. Louis, MO, USA). After synchronized exposure time to DAB, slides were counterstained with 0.1% methyl green (Sigma, St. Louis, MO, USA) at 60 °C for 3 min, dehydrated in ethanol, cleared in xylene, and then mounted with Permount (Fisher Scientific, Pittsburgh PA). Images were obtained at 40× using a Leica DMI4000B confocal microscope with a Retiga 2000R digital camera.

8-OHdG staining was semiquantitatively assessed. The relative staining intensity was measured by color intensity and the percentage of staining tumor cell. The staining color was stratified with three scales, and the staining cell percentage was measured with two scales. The product was subsequently divided into 3 scales (Fig. 2).

### Statistical analysis

The association of survival with adjuvant chemotherapy regime, courses, and therapeutic response were assessed by the Cox proportional hazards model. A multivariate model was then constructed with stepwise regression techniques. 8-OHdG concentrations in leukocyte DNA were positively skewed. In all cases, *p*-value <0.05 was considered statistically significant. Survival distributions were estimated by the Kaplan–Meier method, and statistical significance was determined by log-rank test. Optimal IDS-related potential factors were explored by logistic regression analysis. All data manipulation and statistical analysis were performed by SPSS software v16 (SPSS for Windows, Rel.16. Chicago, SPSS Inc.).

## Results

### Patient characteristics

The clinicopathological characteristics and clinical management of the recruited cases are shown in Table 1. The median follow-up duration of the survivors was 45.8 months (interquartile range, 38.8 months to 56.9 months) in JICR. The baseline CA-125 level in the JICR patients was nearly identical to those of the MDACC patients (Additional file 1: Table S1). The body mass index of the elderly patients was lower than that of the younger cases in both JICR (*p* = 0.04) and MDACC (*p* = 0.03) populations. The frequency of pathological high-grade tumor in the elderly group was higher than that in the younger patients in both JICR (*p* = 0.02) and MDACC (*p* = 0.00) groups. The major ethnicities were Caucasoid, Black, and Hispanic in the MDACC population, but Eastern Asian in the JICR group. The Eastern Cooperative Oncology Group performance status (PS) ≥ 1 was more common in the elderly than in the younger patients in both JICR (*p* = 0.03) and MDACC (*p* = 0.00) patients. Primary treatment differed between the elderly and younger patients from JICR (*p* = 0.00). Elderly patients more commonly recurred within 12 months than younger counterparts in the MDACC group (*p* = 0.04).

**Table 1:** Clinicopathologic characteristics of serous ovarian cancer

Characteristic	≥ 65 years N (%)	< 65 years N (%)	*p* ^*#*^
Baseline CA-125 (U/mL, meida, range)	960 (7–34,000)	840 (7–30,900)	0.55
Body Mass Index (mean SD)	24.1 (3.1)	25.4 (3.8)	0.04
Grade			
High	106 (96.4)	178 (87.3)	0.02
Low	4 (3.6)	26 (12.7)	
FIGO* stage			
I	3 (2.7)	5 (2.5)	0.84
II	8 (7.3)	10 (4.9)	
III	75 (68.2)	146 (71.6)	
IV	24 (21.8)	43 (21.1)	
ECOG PS*			
0	44 (40.0%)	104 (51.0%)	0.03
1	47 (42.7%)	83 (40.7%)	
≥ 2	19 (17.3%)	17 (8.3%)	
Primary treatment			
NAC*	31 (28.2%)	30 (14.7%)	0.00
PDS	50 (45.5%)	151 (74.0%)	
Chemotherapy only	18 (16.4%)	8 (3.9%)	
No Treatment	11 (10.0%)	15 (7.4%)	
Surgical residual^†^			
Optimal	44 (54.3%)	123 (68.0%)	0.04
Suboptimal	33 (40.7%)	48 (26.5%)	
Unknown	4 (5.0%)	10 (5.5%)	
Recurrence free interval (months)			
≤ 12	34 (47.2%)	56 (35.7%)	0.13
> 12	38 (52.8%)	101 (64.3%)	

*p* ^*#*^, Chi-square *P*-value; Baseline^&^, Level at diagnosis; FIGO*, the International Federation of Gynecology and Obstetrics; ECOG PS*, ECOG Eastern Cooperative Oncology Group Performance Status; NAC*, Neoadjuvant chemotherapy; Optimal cytoreduction^†^, the absence of macroscopic disease on the completion of the surgical procedure

**Fig. 1:**
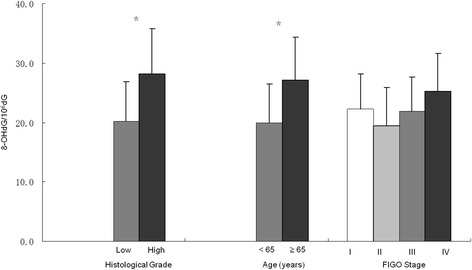
Histological grade and age were associated with mean concentrations of 8-OHdG in leukocyte DNA in serous ovarian carcinoma patients. Statistical significance was calculated using one-way ANOVA testing followed by post hoc analysis. *p < 0.05 vs. subjects with the characteristics in the blank bar

**Fig. 2:**
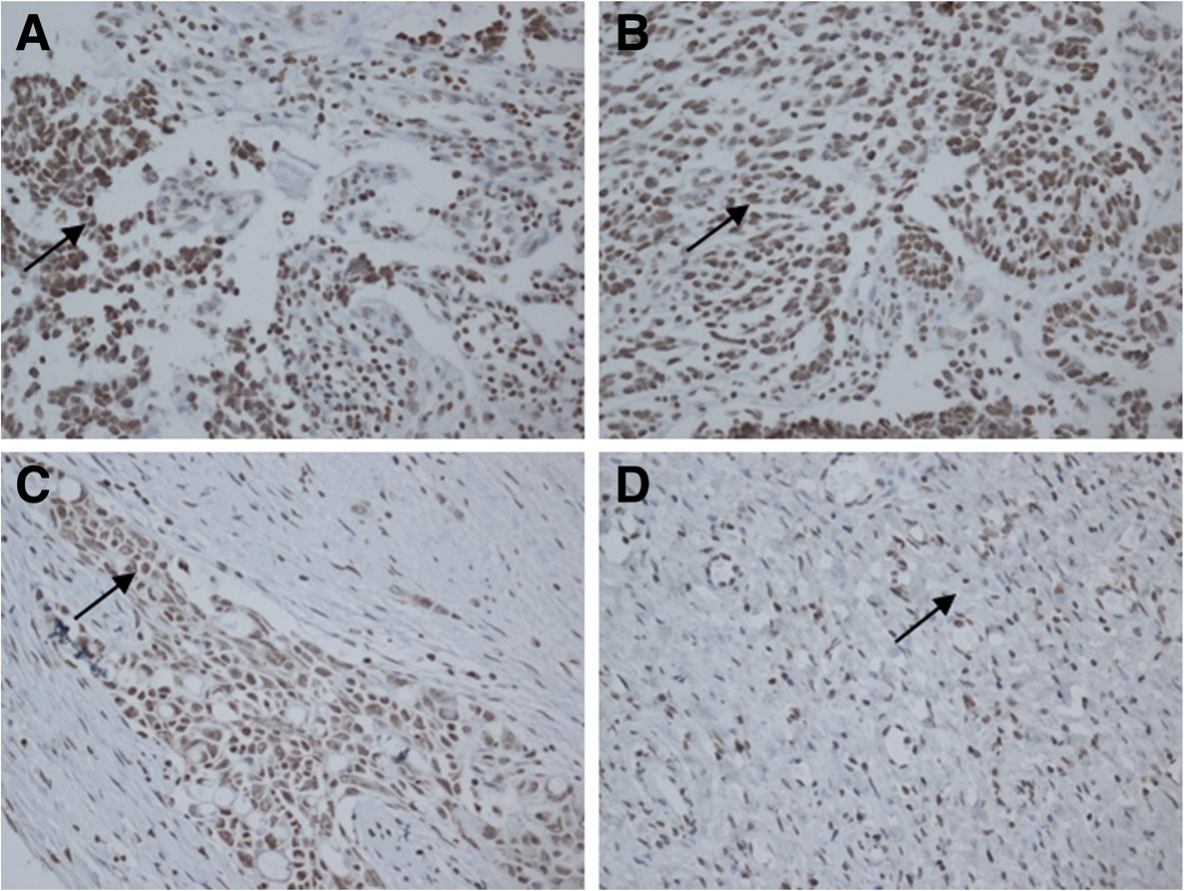
Strong 8-OHdG positive nuclei can be observed in high grade serous ovarian carcinoma. Stromal reaction is negative and cytoplasm is immunopositive in places (**a**). Moderate 8-OHdG positive nuclei can be observed in high grade serous ovarian carcinoma, nuclear immunostaining is also found (**b**). Weak 8-OHdG positive nuclei can also be observed in high grade serous ovarian carcinoma (**c**). Negative control staining for 8-OHdG is also demonstrated in high grade serous ovarian carcinoma (**d**)

The 8-OHdG concentrations in the leukocyte DNA were evaluated in 100 SOC patients and divided into different subgroups by age. The 8-OHdG concentration was higher in the elderly group than in the younger SOCs (27.1 ± 7.2/10^6^ dG vs. 19.9 ± 6.6/10^6^ dG, *p* < 0.01), and was higher in the high grade tumors than in the low grade ones as shown in Fig. 1 (28.2 ± 7.5/10^6^ dG vs. 20.2 ± 6.7/10^6^ dG, *p* < 0.01). We further found that the 8-OHdG expression in SOC tumor was higher in the elderly patients than in the younger cases, but the difference was not significant. There was no statistical difference between the expressions of 8-OHdG in tissue and histological grade (Fig. 2).

### Survival-related factors in serous ovarian cancer

Using the univariate Cox proportional hazards model, we found that PFS and OS were associated with age (*p* = 0.03 and *p* = 0.01, respectively), FIGO stage (*p* < 0.01 and *p* < 0.01, respectively), histological grade (*p* = 0.02 and *p* = 0.01, respectively), baseline CA-125 level (*p* < 0.01 and *p* < 0.01, respectively), and surgery outcome (*p* = 0.02 and *p* = 0.03, respectively) in patients from JICR (Table 2). In the MDACC group, age (*p* < 0.01 and *p* < 0.01, respectively), FIGO stage (*p* = 0.02 and *p* = 0.04, respectively), histological grade (*p* < 0.01 and *p* < 0.01, respectively), baseline CA-125 level (*p* < 0.01 and *p* < 0.01, respectively), and surgery outcome (*p* < 0.01 and *p* < 0.01, respectively) were also associated with PFS and OS (Additional file 2: Table S2).

**Table 2:** Univariate analysis of survival-related factors in serous ovarian cancer

Variable	Progression-free survival (OR, 95% CI)*		Overall survival (OR, 95% CI)*	
Age	1.01	1.00–1.02	1.01	1.00–1.02
FIGO stage				
I	1.00	(reference)	1.00	(reference)
II	2.25	0.85–5.12	1.85	0.70–4.84
III	2.84	1.43–5.95	3.51	1.67–7.58
IV	3.28	1.52–6.87	4.87	2.25–8.83
Grade	1.87	1.25–2.90	2.30	1.68–3.81
Ascites^#^				
< 500 mL	1.00	(reference)	1.00	(reference)
≥ 500 mL	1.75	0.79–6.23	1.15	0.70–5.97
Surgery outcome				
Optimal	1.00	(reference)	1.00	(reference)
Suboptimal	2.97	1.48–7.09	3.54	2.10–5.08
NAC				
Yes	1.00	(reference)	1.00	(reference)
No	1.15	0.74–3.28	1.10	0.70–4.27
Baseline CA-125^†^	1.01	1.00–1.02	1.01	1.00–1.02

OR, 95%CI*, odds ratio, 95% 95% confidence interval

Ascites^#^, ascites volume was estimated by ultrasound at diagnosis

Baseline CA-125^†^, Serum CA-125 concentration at diagnosis

Multivariate analysis revealed that PFS and OS were independently associated with age (*p* = 0.03 and *p* = 0.02, respectively), FIGO stage (*p* = 0.03 and *p* = 0.04, respectively), histological grade (*p* = 0.02 and *p* = 0.02, respectively), and surgery outcome (*p* = 0.04 and *p* = 0.02, respectively) in patients from JICR (Table 3). In the MDACC group, age (*p* < 0.01 and *p* < 0.01, respectively), histological grade (*p* = 0.03 and *p* = 0.02, respectively), and surgery outcome (*p* < 0.01 and *p* < 0.01, respectively) were also associated with PFS and OS (Additional file 3: Table S3).

**Table 3:** Multivariate analysis of survival-related factors in serous ovarian cancer

Variable	Progression-free survival (OR, 95% CI)		Overall survival (OR, 95% CI)	
Age	1.02	1.00–1.02	1.02	1.00–1.02
FIGO stage	1.33	1.12–1.63	1.49	1.29–1.75
Grade	1.61	1.10–2.59	1.88	1.35–2.90
Baseline CA-125	1.00	1.00–1.02	1.01	1.00–1.01
Surgery outcome				
Optimal	1.00	(reference)	1.00	(reference)
Suboptimal	1.25	1.08–1.51	1.58	1.29–1.85
Ascites				
< 500 mL	1.00	(reference)	1.00	(reference)
≥ 500 mL	1.04	0.75–2.05	1.05	0.59–2.72
NAC				
Yes	1.00	(reference)	1.00	(reference)
No	1.02	0.79–1.54	1.03	0.73–1.82

### 8-OHdG level associated with the prognosis of SOC patients who achieved clinical complete response (CCR)

**Fig. 3:**
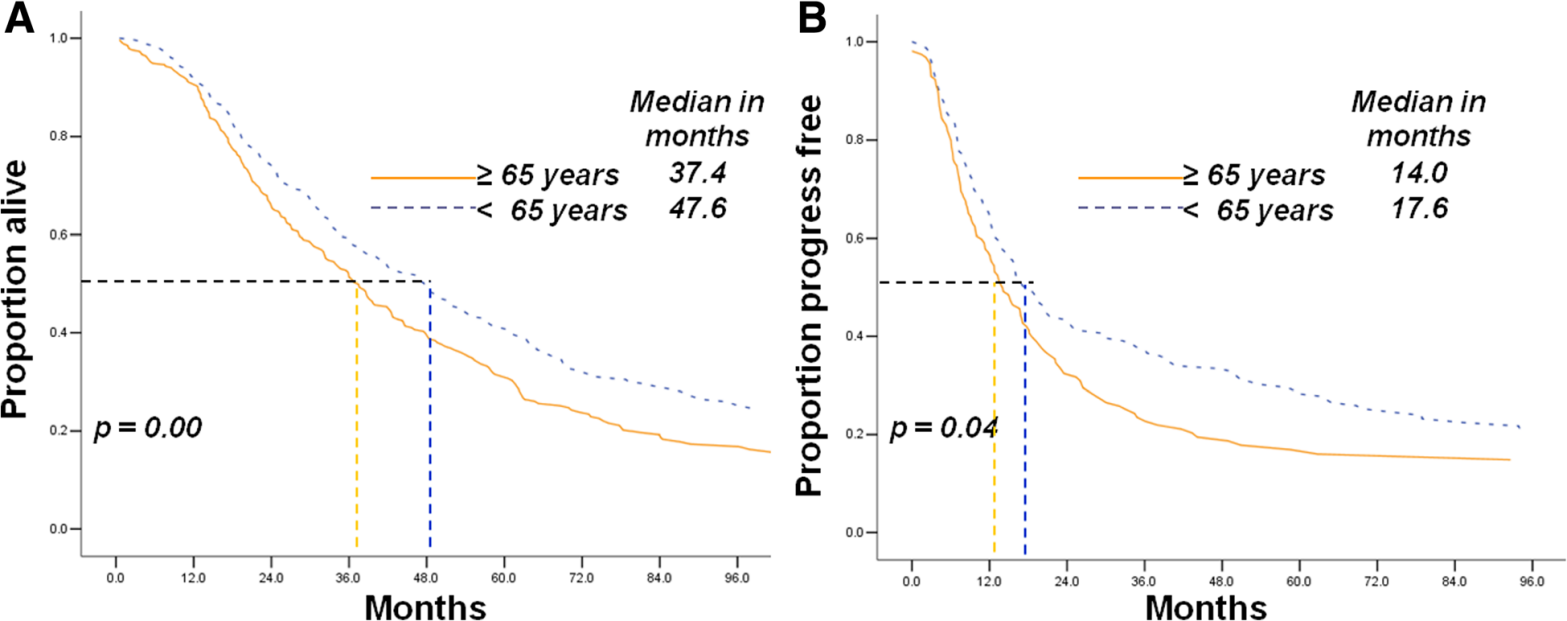
Elderly patients had shorter overall survival and progression-free survival than younger cases with serous ovarian carcinoma from JICR (**a**, **b**)

Using the log-rank test, we observed that the younger patients still exhibited longer OS durations (47.6 months, 95% confidence interval [CI] 41.5–53.7 vs. 37.4 months, [CI] 32.2–42.7; Fig. 3a) and PFS (17.6 months, [CI] 14.5–20.6 vs. 14.0 months, [CI] 10.8–17.3; Fig. 3b) than those of the elderly counterparts with SOCs in the JICR population. This result was confirmed by the data from MDACC, which showed the poorer OS (49.2 months, [CI] 43.5–54.9 vs. 40.8 months, [CI] 36.5–45.1; Additional file 4: Figure S1A) and PFS (18.7 months, [CI] 15.5–21.9 vs. 15.0 months, [CI] 10.1–20.0, Additional file 4: Figure S1B) of the elderly patients than those of the younger patients.

**Fig. 4:**
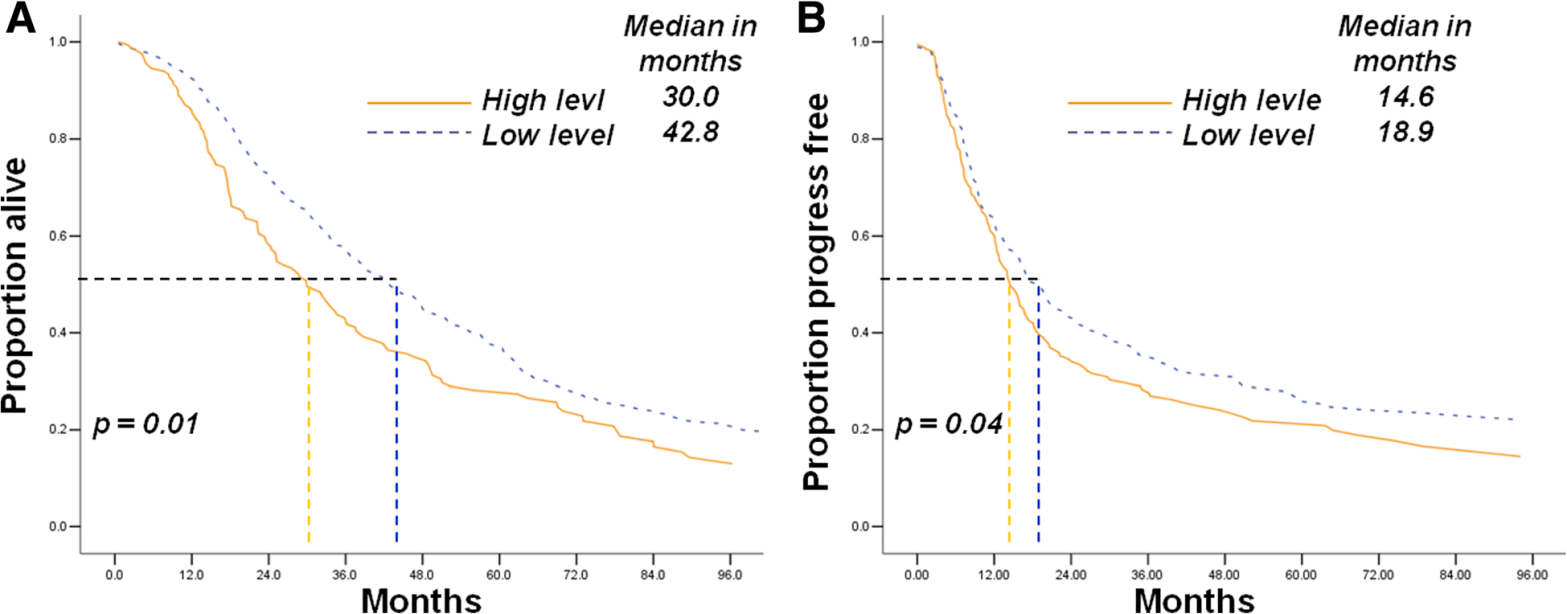
Increasing 8-OHdG concentration in leukocyte DNA is associated with poor prognosis of serous ovarian carcinoma patients received compete clinical response. **a** Low 8-OHdG concentration was associated with longer overall survival duration (42.8 vs. 30.0 months, *p* = 0.01); **b** Low 8-OhdG concentration was associated with longer progression-free survival duration (18.9 vs.14.6 months, *p* = 0.04)

The median OS and PFS durations of the patients with high 8-OHdG concentrations in leukocyte DNA (30.0 months, [CI] 23.5–36.5 and 14.6 months, [CI] 11.9–17.2,) were poorer than those with lower 8-OHdG concentration in the SOC population (42.8 months, [CI] 38.3–47.2 and 18.9 months, [CI] 14.4–23.4, respectively; Fig. 4).

## Discussion

The incidence of EOC increases with age and causes the most deaths in gynecological oncology. The effect of age on prognosis can be analyzed by identifying the clinicopathological characteristics between elderly and younger patients with EOC. However, the clinical and pathological features of elderly patients with EOC have been rarely reported in China or other Asian countries. Most previous reports were limited to cases in Western countries and the USA. Studies have reported that the annual incidence of ovarian cancer varies with race [8, 40, 41]. The genetic background and heredity of patients may be associated with different frequencies of histological subtypes and FIGO stage and thus influence prognosis. Ethnicity must be considered when the association between age and prognostic factors is evaluated. In our study, the clinical features of Chinese patients with SOC were stratified into younger and advanced age groups. The results may indicate the unique features of eastern Asian populations. A validation group was also obtained from MDACC with sources of different ethnicities.

Consistent with previous results [42], our data showed that the elderly patients with SOC constituted more than 30% of the patients from JICR and MDACC populations. More than 90% elderly patients were in FIGO stages III–IV in both groups. Moreover, the high grade and poor PS were more common in the elderly patients from both centers, although these parameters are challenged by some other studies [43–46]. Another significant finding in the management of elderly patients was the higher exposure to NAC than to primary debulking surgery [47, 48].

Conservative therapeutic approaches, including inferior surgical debulking or decreased exposure to standard chemotherapy, have been demonstrated to be associated with short survival duration in elderly patients with EOC [49–53]. In the present study, the Cox proportional hazards model confirmed that advanced age could be an inferior survival factor. A previous study reported that age is an independent prognostic indicator [25], although this finding has been contradictory with those observed in other studies [15, 24]. Our result implies that age may be associated with some inherent adverse biological factors. We found that 8-OHdG concentration in leukocyte DNA was higher in the elderly patients with SOC than in the younger cases. When the influence of different distributions of histological grade was ruled out and the effects of treatment difference with age were minimized, high 8-OhdG levels were found to be associated with short survival durations in patients with SOC who achieved CCR. The dysfunction in 8-OhdG production has been confirmed to be associated with drug resistance and may explain the poor prognosis in the elderly SOC patients [35].

The present study is characterized by several limitations. First, selection biases are unavoidable and inherent to the retrospective nature of our study. In the 20-year duration of the study, new chemotherapy regimens and molecular target agents have emerged [54], various treatment protocols, such as different types of surgery and medication administration programs, have been developed, and disease evaluation methods have been improved. Hence, these developments may have affected the results. We attempted to overcome this shortcoming by performing multivariate and stratified analyses. The relatively stringent recruitment criteria may also partially eliminate the influence of selected factors. A prospective trial with age as a variable is the most appropriate methodology to address the issue in question. Nevertheless, we believe that our results are clinically relevant. Second, the absence of a unified standard for elderly EOC and limited sample size also generated bias, although MDT was established to guide therapy. Lastly, the criterion of “elderly” EOC is unclear because the definition varied from 40 years to 80 years in previous reports. Therefore, our results cannot be directly translated to all elderly ovarian cancer cases until further studies with broader inclusion criteria become available.

## Conclusion

Ederly SOC patients were more commonly diagnosed with poor performance status than their younger counterparts. As such, elderly SOC patients were undertreated. High 8-OHdG concentration in leukocyte DNA was associated with advanced age and poor prognosis in SOC patients.

## Additional files

Additional file 1: Table S1. Clinicopathologic characteristics of serous ovarian cancer from MDACC. (DOCX 14 kb)

Additional file 2: Table S2. Univariate analysis of survival-related factors in serous ovarian cancer from MDACC. (DOCX 13 kb)

Additional file 3: Table S3. Multivariate analysis of survival-related factors in serous ovarian cancer from MDACC. (DOCX 12 kb)

Additional file 4: Figure S1. Elderly patients had shorter overall survival and progression-free survival than younger cases with serous ovarian carcinoma from MDACC (A, B). (BMP 2246 kb)

## Abbreviations

EOC: Epithelial ovarian cancer
IDS: Interval cytoreduction surgery
NAC: Neoadjuvant chemotherapy
OS: Overall survival
PDS: Primary debulking surgery
PFS: Progression-free survival
