# Supplementary material for: Age is associated with prognosis in serous ovarian carcinoma
Source: J Ovarian Res. 2017 Jun 12;10:36. doi: 10.1186/s13048-017-0331-6 (PMC5469143; doi:10.1186/s13048-017-0331-6)
Supplement: Supplementary file 1 — Clinicopathologic characteristics of serous ovarian cancer from MDACC. (DOCX 14 kb) [file 13048_2017_331_MOESM1_ESM.docx]

**Table S1. Clinicopathologic characteristics of serous ovarian cancer from MDACC**

| Characteristic | ≥ 65 years N (%) | < 65 years N (%) | *p^#^* |
| --- | --- | --- | --- |
| Baseline CA-125 (U/mL, meida, range) | 957 (7-31993) | 850.5 (7-33432) | 0.91 |
| Body Mass Index (mean SD) | 26.3 (5.07) | 27.6 (6.55) | 0.03 |
| Ethnicity |  | | |
| Caucasic | 214 (79.3) | 403 (80.0) | 0.99 |
| Black | 12 (4.4) | 24 (4.8) |  |
| Hispanic | 36 (13.3) | 56 (11.1) |  |
| Eastern Asia | 5 (1.9) | 16 (3.2) |  |
| Others^ | 3 (1.0) | 5 (1.0) |  |
| Grade |  | | |
| High | 260 (96.3) | 445 (88.3) | 0.00 |
| Low | 10 (3.7) | 59 (11.7) |  |
| FIGO* stage |  | | |
| I | 6 (2.2) | 15 (3.0) | 0.23 |
| II | 16 (5.9) | 15 (3.0) |  |
| III | 190 (70.4) | 362 (71.8) |  |
| IV | 58 (21.5) | 112 (22.2) |  |
| ECOG PS* |  | | |
| 0 | 116 (43.0%) | 254 (50.4%) | 0.00 |
| 1 | 108 (40.0%) | 207 (41.1%) |  |
| ≥ 2 | 46 (17.0%) | 43 (8.5%) |  |
| Primary treatment |  | | |
| NAC* | 23 (8.5%) | 29 (5.8%) | 0.08 |
| PDS | 214 (79.3%) | 455 (90.3%) |  |
| Chemotherapy only | 14 (5.2%) | 10 (2.0%) |  |
| No Treatment | 19 (7.0%) | 10 (2.0%) |  |
| Surgical residual^†^ |  | | |
| Optimal | 150 (63.3%) | 339 (70.0%) | 0.04 |
| Suboptimal | 86 (36.3%) | 136 (28.1%) |  |
| Unknown | 1 (0.4%) | 9 (1.9%) |  |
| Recurrence free interval (months) |  | | |
| ≤12 | 85 (44.7%) | 138 (35.7%) | 0.04 |
| >12 | 105 (55.3%) | 249 (64.3%) |  |

*p^#^*, Chi-square P-value; Baseline^&^, Level at diagnosis; Others^, including 5 Middle Eastern, and 3 Indian cases.; FIGO*, the International Federation of Gynecology and Obstetrics ; ECOG PS*, ECOG Eastern Cooperative Oncology Group Performance Status; NAC*, Neoadjuvant chemotherapy; Optimal cytoreduction^†^, the absence of macroscopic disease on the completion of the surgical procedure
