# Supplementary material for: Age is associated with prognosis in serous ovarian carcinoma
Source: J Ovarian Res. 2017 Jun 12;10:36. doi: 10.1186/s13048-017-0331-6 (PMC5469143; doi:10.1186/s13048-017-0331-6)
Supplement: Supplementary file 2 — Univariate analysis of survival-related factors in serous ovarian cancer from MDACC. (DOCX 13 kb) [file 13048_2017_331_MOESM2_ESM.docx]

**Table S2. Univariate analysis of survival-related factors in serous ovarian cancer from MDACC**

| Variable | Progression-free survival  (OR, 95% CI)^*^ | |  | Overall survival  (OR, 95% CI)^*^ | |
| --- | --- | --- | --- | --- | --- |
| Age | 1.01 | 1.00-1.03 |  | 1.02 | 1.00-1.03 |
| FIGO stage |  | |  |  | |
| I | 1.00 | (reference) |  | 1.00 | (reference) |
| II | 2.07 | 0.89-4.81 |  | 1.79 | 0.75-4.29 |
| III | 2.77 | 1.37-5.62 |  | 3.31 | 1.57-6.99 |
| IV | 3.11 | 1.48-6.56 |  | 4.40 | 2.06-7.42 |
| Grade | 1.80 | 1.23-2.63 |  | 2.15 | 1.53-3.02 |
| Ascites^#^ |  | |  |  | |
| < 500mL | 1.00 | (reference) |  | 1.00 | (reference) |
| ≥ 500mL | 1.88 | 0.82-5.66 |  | 1.22 | 0.71-5.43 |
| Surgery outcome |  | |  |  | |
| Optimal | 1.00 | (reference) |  | 1.00 | (reference) |
| Suboptimal | 2.95 | 1.44-6.86 |  | 3.46 | 1.99-4.87 |
| NAC |  | |  |  | |
| Yes | 1.00 | (reference) |  | 1.00 | (reference) |
| No | 1.29 | 0.86-2.80 |  | 1.18 | 0.84-2.68 |
| Baseline CA-125^†^ | 1.00 | 1.00-1.02 |  | 1.01 | 1.00-1.01 |

OR, 95%CI^*^, odds ratio, 95% 95% confidence interval

Ascites^#^, ascites volume was estimated by ultrasound at diagnosis.

Baseline CA-125^†^, Serum CA-125 concentration at diagnosis
