# Supplementary material for: Age is associated with prognosis in serous ovarian carcinoma
Source: J Ovarian Res. 2017 Jun 12;10:36. doi: 10.1186/s13048-017-0331-6 (PMC5469143; doi:10.1186/s13048-017-0331-6)
Supplement: Supplementary file 3 — Multivariate analysis of survival-related factors in serous ovarian cancer from MDACC. (DOCX 12 kb) [file 13048_2017_331_MOESM3_ESM.docx]

**Table S3. Multivariate analysis of survival-related factors in serous ovarian cancer from MDACC**

| Variable | Progression-free survival  (OR, 95% CI) | |  | Overall survival  (OR, 95% CI) | |
| --- | --- | --- | --- | --- | --- |
| Age | 1.01 | 1.00-1.02 |  | 1.01 | 1.00-1.02 |
| FIGO stage | 1.31 | 1.10-1.57 |  | 1.46 | 1.26-1.69 |
| Grade | 1.57 | 1.06-2.33 |  | 1.84 | 1.30-2.62 |
| Baseline CA-125 | 1.00 | 1.00-1.02 |  | 1.01 | 1.00-1.01 |
| Surgery outcome |  | |  |  | |
| Optimal | 1.00 | (reference) |  | 1.00 | (reference) |
| Suboptimal | 1.37 | 1.15-1.63 |  | 1.53 | 1.24-1.78 |
| Ascites |  | |  |  | |
| < 500mL | 1.00 | (reference) |  | 1.00 | (reference) |
| ≥ 500mL | 1.08 | 0.85-2.17 |  | 1.10 | 0.79-3.05 |
| NAC |  | |  |  | |
| Yes | 1.00 | (reference) |  | 1.00 | (reference) |
| No | 1.04 | 0.84-1.69 |  | 1.05 | 0.88-1.46 |
