# Supplementary figures and images for: Age is associated with prognosis in serous ovarian carcinoma
Source: J Ovarian Res. 2017 Jun 12;10:36. doi: 10.1186/s13048-017-0331-6 (PMC5469143; doi:10.1186/s13048-017-0331-6)

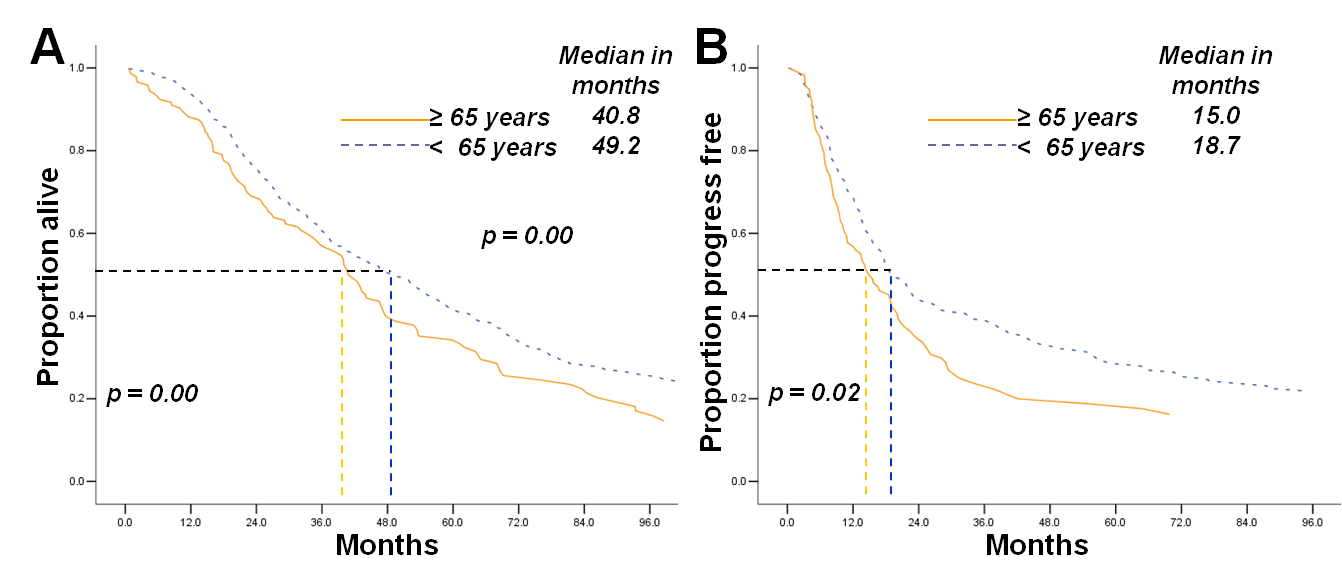

Supplement: Supplementary file 4 — Elderly patients had shorter overall survival and progression-free survival than younger cases with serous ovarian carcinoma from MDACC (A, B). (BMP 2246 kb) [file 13048_2017_331_MOESM4_ESM.bmp]
